# Vision loss due to uncommon “red eye”: A case report

**DOI:** 10.1097/MD.0000000000031064

**Published:** 2022-10-14

**Authors:** Tang Xu-yuan, Qi Rui-nan, Wu Ze-yong, Li Juan, Xie Tao, Yuan Yi-qun

**Affiliations:** a Department of Ophthalmology, The First Affiliated Hospital, School of Medicine, Zhejiang University, China; b Department of Ophthalmology, The Fourth Affiliated Hospital, School of Medicine, Shihezi University, China; c Department of Pathology, The Fourth Affiliated Hospital, School of Medicine, Shihezi University, China.

**Keywords:** case report, hypermature cataract, lens-induced glaucoma, misdiagnosis, phacolytic glaucoma

## Abstract

Phacolytic glaucoma is a rare complication induced by hypermature cataracts and may occasionally be encountered in some rural areas. It tends to be misdiagnosed and induces a risk of permanent vision impairment.

**Patient concerns:**

A 63-year-old Uighur woman complained of redness and decreased vision in her right eye and was treated for endophthalmitis at a primary hospital before being referred to our hospital.

**Diagnosis:**

On admission, the patient had weak light perception in the right eye, an intraocular pressure of 65 mmHg, and slit-lamp examination revealed swelling of the eyelids and significant injection of conjunctiva. The entire cornea was cloudy and edematous, whereas the aqueous humor was milky turbid. Cytological examination of the aqueous liquid confirmed the presence of lens protein-laden macrophages. A Morgagnian cataract was observed after anterior chamber irrigation. So the final diagnosis was phacolytic glaucoma.

**Interventions:**

The patient received anterior chamber irrigation and extracapsular cataract extraction with intraocular lens implantation successively.

**Outcomes:**

Final visual acuity was limited to 6/120 due to secondary optic nerve damage.

**Lessons:**

Phacolytic glaucoma can mimic endophthalmitis and tend to be misdiagnosed, causing permanent vision impairment. Improving awareness of phacolytic glaucoma and enhancing public health education regarding cataracts are ways to prevent phacolytic glaucoma and phacolytic glaucoma-related vision loss.

## 1. Introduction

“Red eye” is one of the most common signs for patients to visit in primary hospitals. Most “red eyes” are not serious, but some of the causes need to be diagnosed and treated quickly. Phacolytic glaucoma (PG) is a rare complication induced by hypermature cataracts that may present as red eye and may occasionally be encountered in basic-level hospitals, especially in rural areas.^[1]^ Here, we report a case of PG with poor visual outcomes, owing to an initial misdiagnosis and final optic nerve damage.

## 2. Case presentation

**Figure 1.**
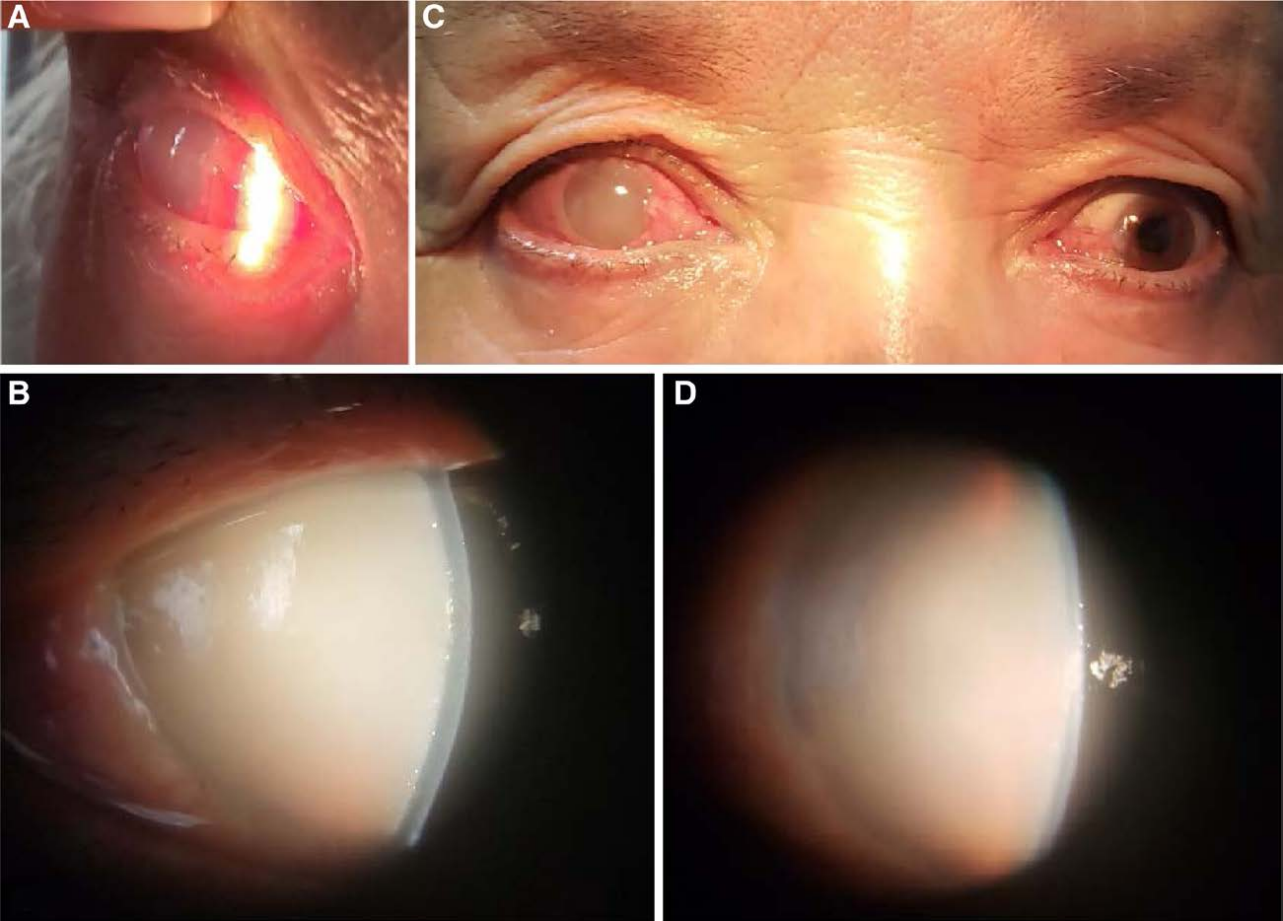
On admission, slit-lamp examination revealed a notable conjunctival injection (Fig. 1a). The entire cornea was cloudy and edematous, the aqueous humor was milky turbid, and no details regarding the iris, pupil, lens or fundus were visible in the right eye (Fig. 1b). Injection of the conjunctiva, corneal edema, and aqueous humor’s turbidity were slightly reduced after IOP lowering and administration of anti-inflammatory medications (Fig. 1c, d). IOP = intraocular pressure.

A 63-year-old Uighur woman presented with a red eye and decreased vision in the right eye for 4 days. The patient visited a primary hospital and was diagnosed with endophthalmitis. The patient was healthy, had no history of ocular trauma or surgery, and denied fever or other systemic symptoms. She had received systemic and topical antibiotics for seven days before being referred to our hospital. On admission, she had weak light perception in the right eye, intraocular pressure (IOP) was 65 mmHg, slit-lamp examination revealed swelling of the eyelids and notable injection of the conjunctiva (Fig. 1a). The entire cornea was cloudy and edematous, whereas the aqueous humor was milky turbid. No details regarding the iris, pupil, lens or fundus were visible in the right eye (Fig. 1b). For the left eye, there were no significant positive findings except for a moderate nucleus, best corrected vision acuity of 20/25, and IOP of 19 mmHg. An ocular B-scan revealed mild vitreous opacity and the absence of other abnormalities in both eyes. The corneal endothelial count was 2448/mm^2^ in the left eye, and no data were available for the right eye.

Based on above-mentioned findings, lens induced glaucoma came first, but the diagnosis was not certain, since the lens of the affected eye was invisible, while the cataract in the fellow eye was far from “hypermature”. Unfortunately, there was no ultrasound biomicroscopy in our department, while anterior segment optical coherence tomography and gonioscopy examination failed to show the anterior chamber (AC) angle of the affected eye.

**Figure 2.**
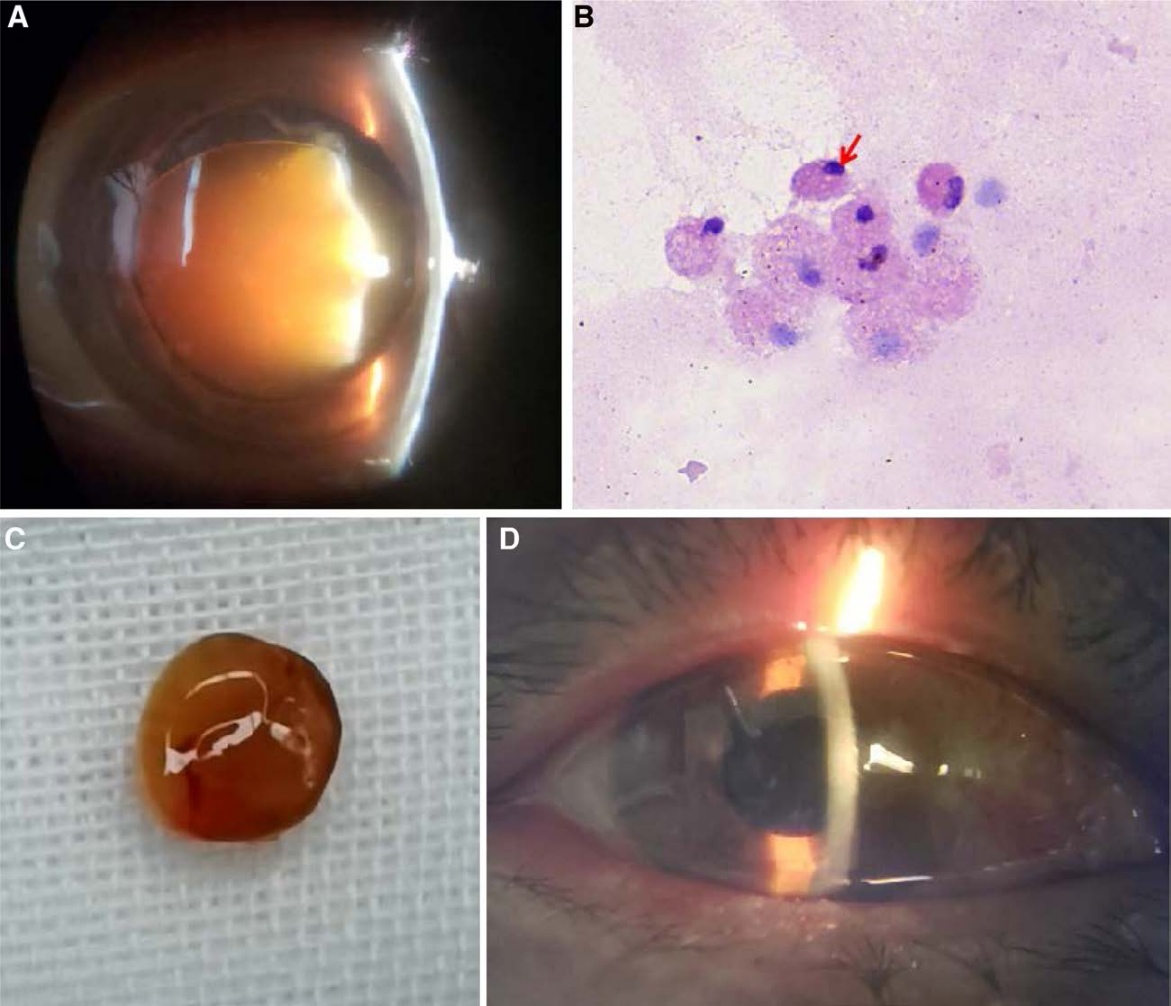
A Morgagnian cataract was observed after anterior chamber irrigation (Fig. 2a). Cytological examination of the aqueous liquid confirmed the presence of lens protein-laden macrophages (red arrow) (hematoxylin and eosin staining, 400× magnification) (Fig. 2b). The hard nucleus was extracted intact during extracapsular cataract extraction and IOL implantation process (Fig. 2c). One month after cataract surgery, the cornea and aqueous liquid were clear, the intraocular lens was in the capsule bag with a slight enlarged pupil (Fig. 2d). IOL = intraocular lens.

She received intravenous mannitol, cartels and alphagan eye drops to control the IOP, tobradex, and pranopulin eyedrops were also applied to alleviate the ocular inflammation, the cornea’s edema was slightly reduced (Fig. 1c, d). When IOP was below 30 mmHg, she underwent AC irrigation on the 7th day. A Morgagnian cataract was visualized after irrigation (Fig. 2a). Milky aqueous liquid was collected for cytological examination, which confirmed the presence of lens protein-laden macrophages (Fig. 2b), so the diagnosis of PG was confirmed. The IOP remained over 40 mmHg after irrigation; therefore, medications and daily paracentesis were performed for another 5 days before cataract surgery. Since the capsule of the lens was loose and the nucleus was extremely hard, extracapsular cataract extraction and intraocular lens implantation (ECCE + IOL) were performed and the nucleus was extracted in an intact manner (Fig. 2c).

The cornea became clear and IOP was steadily controlled after cataract surgery. However, the extent of the vision improvement was limited. One month after surgery, the best-corrected visual acuity was 6/120, IOP was 11 mmHg, and intraocular lens was in the capsular bag (Fig. 2d). Fundus examination revealed a pale optic disc, and the visual evoked potential showed a significant decrease in optic nerve conduction in the right eye.

## 3. Discussion and conclusions

The pathogenesis of PG involves leakage of high-molecular-weight proteins through the anterior lens capsule, leading to an immunologic reaction and macrophage activity. Protein-rich macrophages and inflammatory debris not only block the trabecular meshwork, resulting in an acutely elevated IOP,^[2]^ but may also be deposited in the inferior chamber, showing a pseudohypopyon appearance^[3,4]^ and mimicking keratitis, endophthalmitis, or uveitis.^[5,6]^

The clinical manifestations of PG include red eye, ocular pain and visual loss, accompanied by headache and other symptoms caused by acute ocular hypertension. PG can be diagnosed mainly based on clinical features, the key defining ones includes elevated IOP with open angle, the presence of macrophages in aqueous solution, and an intact lens’ capsule, which might exhibit dehiscences or holes upon electron microscopic examination.^[3]^

Owing to advances in technology and better outreach programs, cataract surgery is performed considerably earlier, so hypermature cataracts and induced complications are rarely encountered in the current phaco era.^[1]^ Hence, clinicians and ophthalmologists may have insufficient knowledge regarding this disease, contributing to the difficulty in making a quick and definite diagnosis of PG.

The sudden onset of “red eye” and ocular pain often compels patients with PG to seek medical help. As long as the diagnosis is correct and the treatments are timely, further corneal and optic nerve damage can be prevented and good vision will be achieved after cataract surgery. However, if primary clinicians lack awareness of this entity, the outcome might be far from ideal.

Generally, the degree of cataract in both eyes is symmetrical, therefore, when the lens of the affected eye is invisible, an advanced cataract or pseudophakia state in the lateral eye may provide hints. Rather, if lens opacity is not consistent in both eyes, the fellow eye’s condition may be misleading, just as in the present case, whose lateral cataract was far from “hypermature”, combined with severely mixed injection and extremely turbid AC humor in the affected eye, leading to an initial hastily ruling out the possibility of lens-induced glaucoma and misdiagnosed as endophthalmitis.^[7]^ Misdiagnosis is linked to inappropriate treatment and subsequent optic nerve damage, resulting in permanent visual impairment.

It is known that endophthalmitis usually has a history of trauma or eye surgery, or is accompanied by signs of systemic infection, IOP is normal or moderately elevated, hypopyon might be detected in the AC or vitreous cavity, lens opacity can be of various degrees, and retinal necrotic lesions may be revealed when the fundus is detectable. All these signs are different from those of PG. However, when the AC is too cloudy to detect the intraocular details, it would generate difficulties in identifying the cause. In this case, ultrasound biomicroscopy and B-scan might be helpful. Microscopic examination of aspirated aqueous is not always required, but can facilitate the identification of suspected PG patients.^[5]^

Cataract extraction is a definitive treatment for PG but should not be conducted until IOP is controlled. Stable and normal IOP is important to prevent intraoperative complications such as suprachoroidal effusions and expulsive hemorrhage, which may occur due to sudden decompression of the globe. Combined anti-inflammatory therapy is recommended before surgery. When IOP is under control and inflammation is reduced, AC irrigation and cataract surgery can be performed together or successively, depending on the transparency of the cornea. In our case, cataract surgery was performed at another selected time for safe reason because of the cornea’s opacity. When it comes to cataract surgery, extracapsular cataract extraction or small-incision cataract surgery might be more suitable for PG patients, since the nucleus is usually extremely hard, with compromised zonular area, both of which would pose a risk of endothelial damage, zonula dialysis, and a posterior capsular tear during the phacoemulsification process.^[8,9]^ In some cases, the capsular bag may come out entirely, which would inevitably necessitates intracapsular cataract surgery.

The poor visual outcome in our case was partly due to the late presentation of the patient and initial misdiagnosis at the primary hospital. However, the underlying causes of PG and PG-related vision loss might be inadequate public health literacy regarding cataract development and relatively poor access to ophthalmic healthcare in remote areas. In some domestic rural areas, it is still popularly believed that a cataract should not be operated upon until it has matured or that individuals should wait until they cannot see. Currently, China, including Xinjiang, has a universal medical insurance system. Financial conditions are no longer the main reason preventing people from seeking medical care, but lacking of access and knowledge is. Similar situations may also occur in other developing countries.^[10–12]^

To prevent PG and PG-related visual loss, primary care physicians and ophthalmologists should keep in mind this uncommon “red eye” cause, so as to give correct diagnosis and timely treatments when encountered. Moreover, governments should strengthen health education regarding cataracts and improve eye medical resources in remote areas.

## Acknowledgements

We thank TopEdit (www.topeditsci.com) for its linguistic assistance during the preparation of this manuscript.

## Author contributions

**Conceptualization:** Tang Xuyuan.

**Data curation:** Qi Ruinan, Li Juan, Xie Tao.

**Funding acquisition:** Tang Xuyuan.

**Investigation:** Tang Xuyuan, Wu Zeyong, Yuan Yiqun.

**Methodology:** Tang Xuyuan.

**Project administration:** Wu Zeyong.

**Resources:** Tang Xuyuan.

**Supervision:** Tang Xuyuan.

**Validation:** Xie Tao.

**Writing – original draft:** Tang Xuyuan, Qi Ruinan, Xie Tao, Li Juan.

**Writing – review & editing:** Tang Xuyuan, Wu Ze-yong.
